# HDAC inhibitors attenuate the development of hypersensitivity in models of neuropathic pain

**DOI:** 10.1016/j.pain.2013.05.021

**Published:** 2013-09

**Authors:** Franziska Denk, Wenlong Huang, Ben Sidders, Angela Bithell, Megan Crow, John Grist, Simone Sharma, Daniel Ziemek, Andrew S.C. Rice, Noel J. Buckley, Stephen B. McMahon

**Affiliations:** a King’s College London, Wolfson Centre for Age-Related Diseases, London SE1 1UL, United Kingdom; b Pain Research Group, Department of Surgery and Cancer, Imperial College London, London SW10 9NH, United Kingdom; c Neusentis, Pfizer Worldwide R&D, Cambridge CB21 6GS, United Kingdom; d King’s College London, Institute of Psychiatry, Centre for the Cellular Basis of Behaviour, London SE5 9NU, United Kingdom; e UCL Genomics, UCL Cancer Institute and Wolfson Institute for Biomedical Research, London WC1E 6BT, United Kingdom; f Computational Sciences Center of Emphasis, Pfizer Worldwide Research and Development, Cambridge, MA, United States

**Keywords:** Histone deacetylase, Histone deacetylase inhibitors, Neuropathic pain

## Abstract

Histone deacetylase inhibitors (HDACIs) interfere with the epigenetic process of histone acetylation and are known to have analgesic properties in models of chronic inflammatory pain. The aim of this study was to determine whether these compounds could also affect neuropathic pain. Different class I HDACIs were delivered intrathecally into rat spinal cord in models of traumatic nerve injury and antiretroviral drug–induced peripheral neuropathy (stavudine, d4T). Mechanical and thermal hypersensitivity was attenuated by 40% to 50% as a result of HDACI treatment, but only if started before any insult. The drugs globally increased histone acetylation in the spinal cord, but appeared to have no measurable effects in relevant dorsal root ganglia in this treatment paradigm, suggesting that any potential mechanism should be sought in the central nervous system. Microarray analysis of dorsal cord RNA revealed the signature of the specific compound used (MS-275) and suggested that its main effect was mediated through HDAC1. Taken together, these data support a role for histone acetylation in the emergence of neuropathic pain.

## Summary

Intrathecal delivery of histone deacetylase inhibitors ameliorates hypersensitivity in models of neuropathic pain. This effect may be mediated at the level of the spinal cord through inhibition of HDAC1 function.

## 1 Introduction

Although chronic neuropathic pain remains an area of considerable unmet clinical need [36], our view of the underlying pathology has shifted considerably over the past 2 decades. It was initially considered a neurophysiological issue – excessive neuronal activity in specific pathways. Currently, it is more usually discussed in terms of maladaptive plasticity rooted in altered transcriptional expression, and indeed there are now a very large number of examples of such change [38], [43]. Yet, despite a wealth of expression studies, it is still unclear how these processes are coordinated. One possibility is that there is involvement of epigenetic mechanisms, such as histone acetylation.

Acetylation of histone lysine residues, such as lysine residue 9 (H3K9ac), relaxes chromatin structure, recruits and stabilizes chromatin remodeling enzymes, including transcription factors [51], and promotes transcriptional elongation [47]. The process is controlled through the dynamic interplay of histone acetyltransferases (HATs) and histone deacetylase (HDAC) enzymes. HDACs first aroused interest in clinical research when they were discovered to be of importance in cancer, with their inhibition having chemotherapeutic effects [6], [42]. In the nervous system, they have since been shown to be involved in many disease models (eg, of neurodegenerative disorders [15] and depression [19]), as well as in fundamental cellular processes, such as neuronal plasticity [29], [46].

In chronic pain, studies of HDACs and their inhibitors are only just beginning to emerge [20]. Sodium valproate, which interferes with HDACs as well as the GABAergic system, has been shown to be an effective analgesic under some circumstances in rodents [41], [61] and, more controversially, in humans [1], [24]. Pan-HDACIs, such as trichostatin A and vorinostat, are claimed to affect visceral hypersensitivity [58] as well as morphine-related phenomena, such as tolerance-induced hyperalgesia [40] and conditioned place preference [5]. Finally, the most well-studied, and hence perhaps the most convincing paradigm in this context has been to administer HDACIs in models of acute inflammatory pain (complete Freund’s adjuvant or formalin). Three independent groups have reported various class I selective- and pan-HDAC compounds to reduce rodents’ nociceptive responses, whether delivered systemically or intrathecally or into the descending modulatory region of the raphe nuclei [2], [12], [13], [65].

There is evidence from expression studies that histone acetylation could also be relevant in neuropathic pain [35], [65]. However, a clear demonstration that selective interference with HDAC activity can affect this type of chronic pain condition is still lacking. The present study attempted to address this issue using chronic intrathecal delivery of 2 different class I selective HDACIs (MS-275 and MGCD0103) into the spinal cord of rats. Animals were subjected to 1 of 2 traumatic nerve injury models (partial sciatic nerve ligation or L5 spinal nerve transection) or to a more clinically relevant model of antiretroviral drug–induced neuropathy [32]. In all cases, increased histone acetylation was observed in the spinal cord and behavioral hypersensitivity was reduced significantly, indicating that HDACs play a role in the emergence of neuropathic pain conditions.

## 2 Materials and methods

### 2.1 Animals

Adult male Wistar rats were used for all experiments (Charles River, Margate, UK). They were housed under standard conditions (12-hour light/dark cycle, lights on between 7:00 am and 7:00 pm, in groups of 4–6) and were experimentally naive before any testing. At the time of surgery, animals weighed between 220 and 250 g. All treatment and care of animals was in accordance with the United Kingdom Animals Scientific Procedures Act (1986).

### 2.2 Behavioral experiments

At the beginning of all behavioral studies, rats were randomized into groups using a list randomizer (random.org), and any subsequent testing was performed by an experimenter who was blind to the assignment. F.D. and J.G. carried out behavioral tests after nerve injury, whereas W.H. carried out behavior after antiretroviral drug therapy in a different department, controlling to some extent for variables that are known to affect behavioral outcome (experimenter and testing environment) [11]. Animals were only excluded if they died during the surgery or if, on dissection, the surgery was determined to be flawed (ie, L4 spinal nerve transection, instead of L5, occurred in 2 of 48 cases). Behavioral results were not analyzed until after the completion of testing and the application of the dissection exclusion criterion.

#### 2.2.1 Mechanical hypersensitivity

**Fig. 1:**
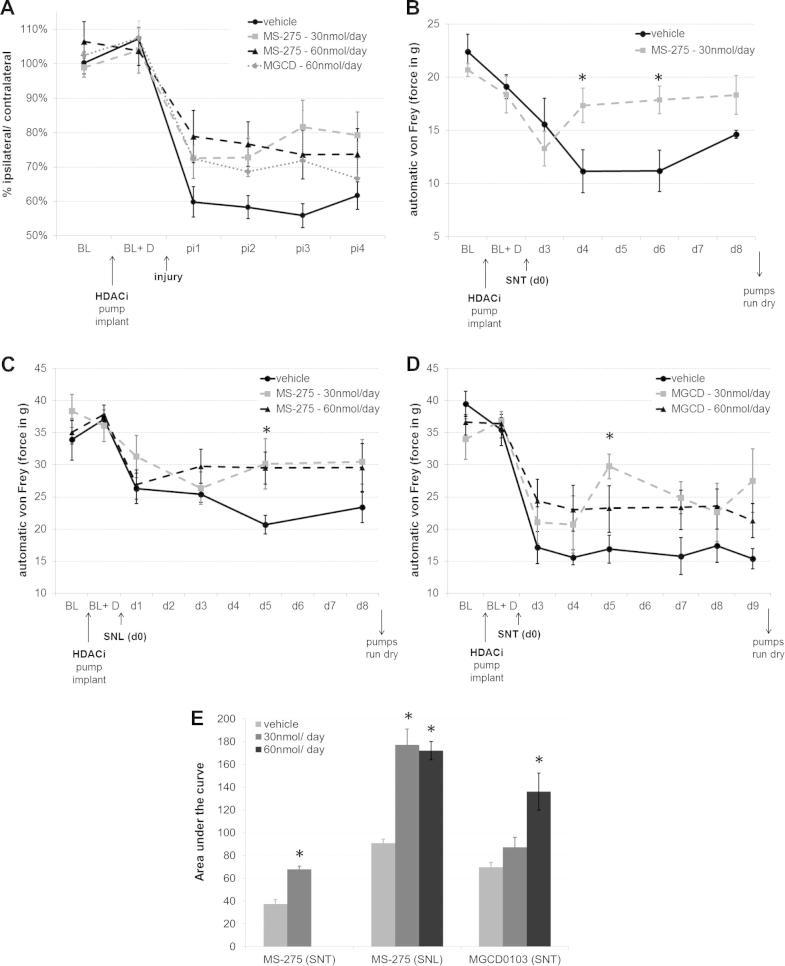
Intrathecal histone deacetylase inhibitor HDACI treatment improved mechanical hypersensitivity after peripheral nerve injury. (A) A summary of the percentage difference between ipsilateral and contralateral paw withdrawal thresholds (in grams) collected across 4 independent experiments (*n* = 36, *n* = 14, *n* = 15, and *n* = 11 for the respective treatment groups). Compared with vehicle, delivery of HDACI (MS-275 at 30 nmol/d, MS-275 at 60 nmol/d, MGDC0103 at 60 nmol/d) significantly reduced withdrawal thresholds (as measured by automatic von Frey) in nerve-injured rats. Some of the raw data are displayed in parts B through D. (B) Intrathecal delivery of MS-275 (30 nmol/d via an osmotic pump) improved mechanical hypersensitivity after L5 spinal nerve transection: repeated-measures analysis of variance (ANOVA) (*n* = 6) with baseline as a covariate; main effect of drug (*F*_1,9_ = 3.73, *P* < .001); interaction between drug × day (*F*_2,18_ = 36.4, *P* = .045). Drug delivery started 5 days before the injury and did not cause any change in baseline withdrawal thresholds (BL + D). (C) MS-275 also significantly increased von Frey withdrawal thresholds after partial sciatic nerve ligation: repeated-measures ANOVA (*n* = 7), main effect of drug (*F*_2,19_ = 3.785, *P* = .041). (D) A different class I HDACI (MGCD0103) had similar analgesic effects after L5 spinal nerve transection. Repeated-measures ANOVA (*n* = 6, vehicle and 60-nmol dose, *n* = 4, 30-nmol dose) with baseline as a covariate, main effect of drug (*F*_2,12_ = 4.902, *P* = .028), interaction between drug × injury (*F*_2,12_ = 4.341, *P* = .038). (E) Area under the curve measurements for the 3 graphs (B–D), with stars indicating significant differences at *P* < .005 (independent samples *t* tests). BL = baseline; BL + D = baseline measured while drugs were delivered intrathecally; pi = post-injury; SNT = L5 spinal nerve transection; SNL = partial sciatic nerve ligation. Error bars indicate SEM. Stars designate individual days on which the difference between drug and vehicle treatment was particularly striking and survived Bonferroni post hoc tests (*P* < .05).

**Fig. 2:**
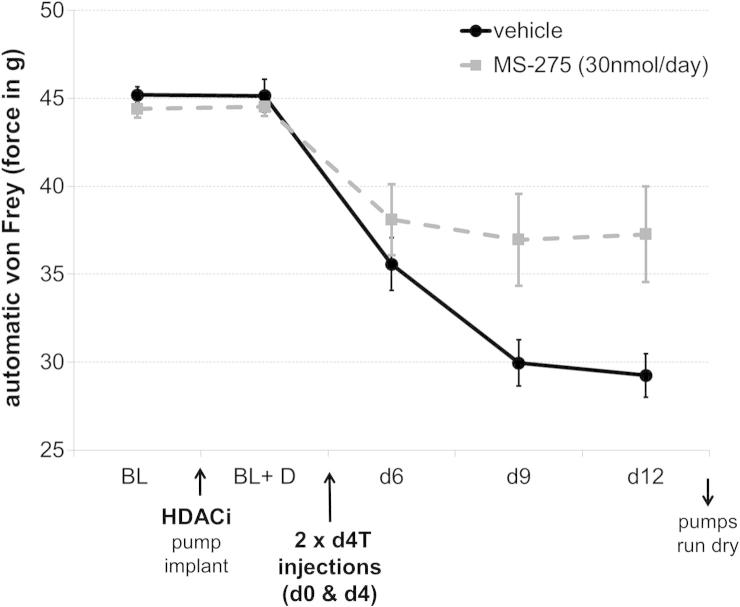
Intrathecal (HDACI) treatment attenuated mechanical hypersensitivity in a more clinically relevant model of antiretroviral (d4T)–induced neuropathy. HDACI pretreatment (MS-275 at 30 nmol/d) was delivered for 7 days, followed by induction of neuropathy using 2 tail vein injections of the antiretroviral drug d4T. The presence of the inhibitor significantly reduced mechanical hypersensitivity: repeated-measures ANOVA (*n* = 7), interaction between day × drug (*F*_5,60_ = 5.002, *P* = .028). Stars designate individual days on which the difference between drug and vehicle treatment was particularly striking and survived Bonferroni post hoc tests (*P* < .05).

A dynamic plantar esthesiometer (Ugo Basile, Milan, Italy) was used to assess mechanical sensitivity thresholds in rats. A probe was applied with increasing force to the plantar surface of each paw (1-50 g, ramping up over 20s), and withdrawal thresholds were measured in triplicate. Before the start of testing, the animals were habituated to the experimenter and the apparatus. Baseline testing was performed twice before implantation of the intrathecal catheter, after which at least 1 further baseline measure was collected while the drug was infused. No change was observed from the baseline before and after drug treatment in healthy animals. After creating the neuropathic lesion, animals were tested at a minimum of 3 time points while the drug was still being infused. Different esthesiometers were used after nerve injury and after d4T, leading to different baseline measurements (Fig. 1 vs Fig. 2), and in the case of nerve injury, the esthesiometer was serviced between completed experiments, again leading to different baselines in Figure 1B vs C and D.

#### 2.2.2 Thermal hypersensitivity

**Fig. 3:**
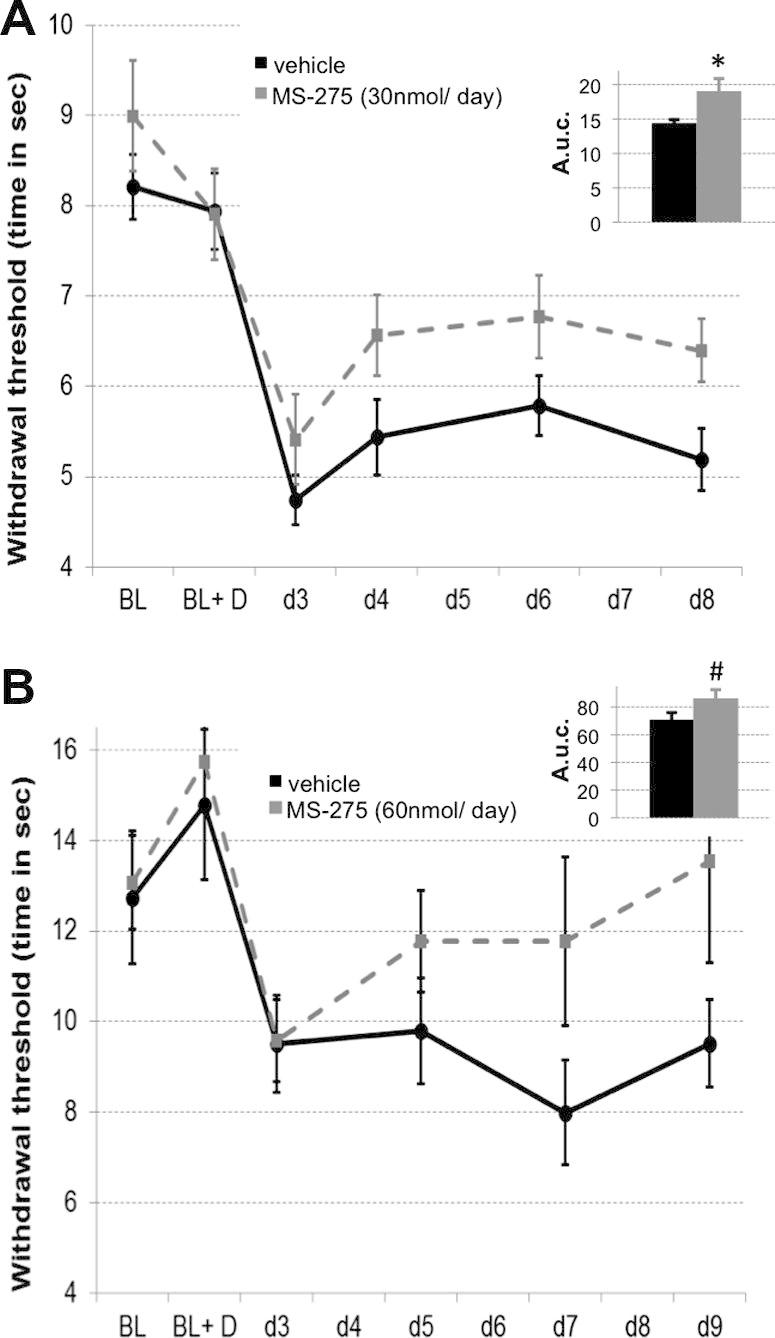
HDACI treatment improved thermal hypersensitivity after neuropathic injury. In 2 separate experiments, pretreatment with intrathecal MS-275 was found to increase withdrawal thresholds after nerve injury on a radiant heat paw withdrawal assay. (A) A 30-nmol/d dose: repeated-measures ANOVA (*n* = 6), main effect of drug (*F*_1,11_ = 5.67, *P* = .036). (B) A 60-nmol/d dose: repeated-measures ANOVA (*n* = 6), main effect of drug (*F*_1,11_) = 6.265, *P* = .029). Insets show area under the curve measurements (independent-sample *t* tests, ^∗^*P* < .05, ^#^*P* = 0.1).

A Hargreaves’ apparatus (Ugo Basile) was used to focus an infrared beam on the plantar surface of each paw, and the time to withdraw was used to assess thermal sensitivity thresholds [31]. Values were collected in triplicate for each paw. The experimental design was as described previously for von Frey testing. The data for the 2 different graphs (Fig. 3A and B) were collected at different times in 2 separate experiments, rendering the direct comparison of absolute baseline measures impossible.

### 2.3 Surgery

Intrathecal catheters connected to Alzet osmotic pumps (flow rate of 0.25 μL/h for 28 days or 0.5 μL/h for 14 days) were implanted in rats under medetomidine hydrochloride (0.25 mg/kg) and ketamine (60 mg/kg) anesthesia. A laminectomy was performed at T10–12 and 2 cm of the catheter was inserted caudal to the opening beneath the dura so that its tip lay at the L2–3 level of the spinal cord. The pump was placed subcutaneously on the animal’s back.

Subsequent nerve injury was performed under isoflurane. For partial sciatic nerve ligations [49], the sciatic nerve was exposed at mid-thigh level, above any branching, and approximately two-thirds were tightly ligated with a 4–0 suture. For L5 spinal nerve transections [16], an incision was made to access L5–6 spinal processes. A third of the L6 transverse process was removed, and the underlying L5 spinal nerve was ligated and transected distal to the suture.

**Fig. 4:**
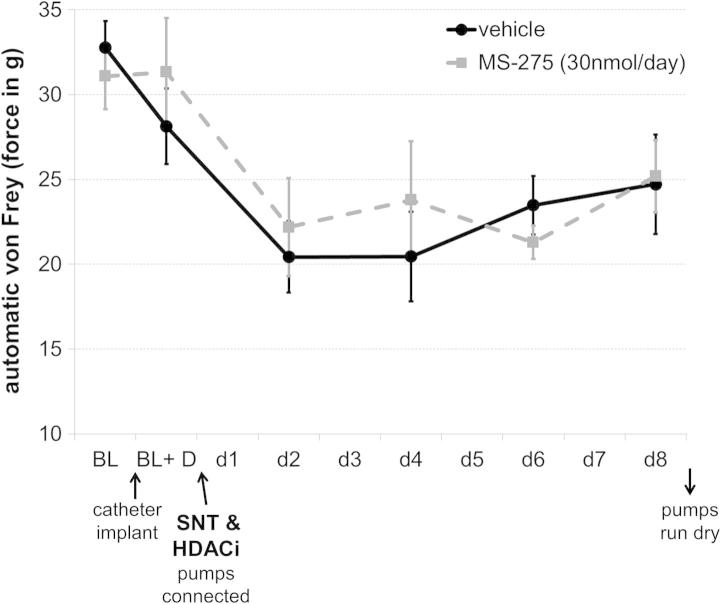
HDACI treatment did not reverse already established hypersensitivity. Pumps were connected immediately after the nerve injury was carried out, resulting in the drug reaching the spinal cord ∼24 hours after the initial insult. No effect on von Frey thresholds was observed with this treatment paradigm. SNT = spinal nerve transaction.

For experiments reported in Figures 1–3, pumps were placed at least 5 days before nerve injury or d4T injections to allow ample pretreatment with HDACI. To test whether HDACIs could rescue already established pain (Fig. 4), the catheter was implanted but left unconnected. Animals were left to recover and tested for any changes in sensitivity threshold. Nerve injury was then carried out, and at the end of the surgery, unprimed Alzet pumps were hooked up to the catheter. According to the pump manufacturer, the drug should take approximately 24 hours to reach the central nervous system without priming.

### 2.4 Antiretroviral-induced neuropathy

Induction of peripheral neuropathy using the antiretroviral drug stavudine (d4T) has been previously described [32]. Briefly, 50 mg/kg d4T (a gift from Pfizer Ltd., made by ZereneX Molecular Limited, Greater Manchester, UK) was delivered intravenously into the tail vein of rats while they were under brief general anesthesia. Two injections were administered, separated by a 4-day gap. Behavioral testing for hind paw mechanical hypersensitivity was carried out 6, 9, and 12 days after the first d4T injection.

### 2.5 Drugs

HDACIs were purchased from Selleck Chemicals (Houston, TX) and administered in Alzet mini-pumps over a period of 2 or 4 weeks (models 2002 or 2004; Charles River Laboratories, Margate, UK). Both MS-275 and MGCD0103 were administered at 30 or 60 nmol/d. The drugs were dissolved in 30% dimethyl sulfoxide (DMSO) in saline solution, which was also used as a vehicle to control for any adverse effects of the DMSO solvent. The relatively high DMSO concentration was required to ensure solubility of the compounds, but would have been diluted on delivery (0.5 or 0.25 μL/h, administered into much larger volumes of cerebral spinal fluid) and has been previously shown to have no effect on nociceptive processing when administered intrathecally [18]. The results presented here were consistent with this: no significant changes in thermal or mechanical sensitivity thresholds were observed between naive rats (baseline, BL) and pump-implanted, uninjured rats (baseline + drug [BL + D]) with vehicle (or indeed HDACI) treatment. Moreover, saline solution or HDACI, both in 30% DMSO, did not affect the contralateral paw at any point (data not shown), and when pumps where implanted after the injury, no significant behavioral changes could be observed before or after nerve transection (Fig. 4).

### 2.6 Dissection

After the behavioral experiments, at day 14 after injury or first d4T injection, rats were killed and spinal cord and DRG tissue was dissected and snap-frozen in liquid nitrogen. Spinal nerves were followed up from their respective ganglia to ensure consistent dissection of spinal cord segments L4–6. The open book method was used to isolate dorsal ipsilateral and contralateral segments.

### 2.7 Western blots

**Fig. 5:**
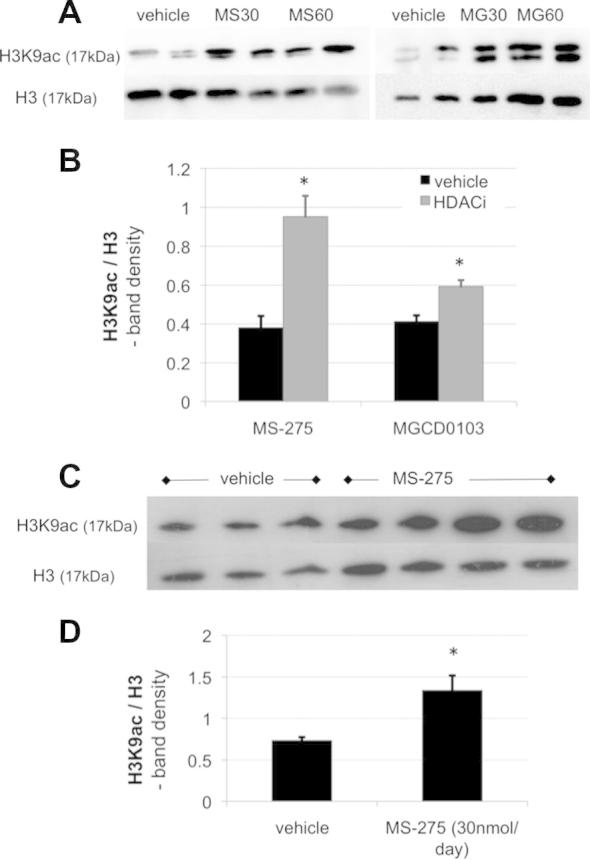
Intrathecal HDACI treatment globally increased acetylation at lysine residue 9 of histone 3 (H3K9ac) in the dorsal spinal cord. Shown are representative Western blots against H3K9ac after vehicle, MS-275 (MS30, MS60 at 30 nmol and 60 nmol/d, respectively) and MGCD0103 treatment (MG30, MG60 at 30 nmol and 60 nmol/d, respectively). Protein was obtained from ipsilateral dorsal spinal cord of animals with neuropathy as a result of spinal nerve transection (A) or antiretroviral drug injection (C). The blots were stripped and reprobed with an antibody against total H3 as a loading control. (B, D) Quantification of Western blot data using band-density analysis in ImageJ software. Significantly increased H3K9 acetylation was observed in the nerve injury model after both MS-275 and MGCD0103 treatment (*n* = 4, independent-sample *t* tests, *P* < .01) and in the drug-induced neuropathy model after MS-275 treatment (*n* = 3, independent-sample *t* test, *P* = .017).

Proteins were homogenized in lysis buffer (2% Empigen, 3 mM DTT, 1 mM sodium orthovanadate, 5 mM sodium fluoride, 5 mM sodium butyrate, 1x protease inhibitors in phosphate-buffered saline) and histones were isolated through acid extraction (0.2 M HCl for 3 hours at 4 °C). Standard sodium dodecyl sulfate–polyacrylamide gel electrophoresis (12–16% gels) and transfer onto 0.45-μM polyvinylidene fluoride membranes (Millipore, Watford, UK) was used for Western blot. Membranes were blocked in 5% milk and incubated overnight at 4 °C with a primary antibody at 1:1000 dilution (rabbit H3K9ac; Abcam, Cambridge, UK or Cell Signaling Technology, Danvers, MA) or rabbit H3 from Abcam). After 3 washes in Tris-buffered saline and 1% Tween, a secondary antibody was used for 1 hour (1:5000, horseradish peroxidase–conjugated anti-rabbit; GE Healthcare, Buckinghamshire, UK). The signal was revealed after further washes using an ECL prime kit (GE Healthcare, Waukesha, WI) and visualized in a UVP GelDoc-It Imaging system (Ultraviolet products, Upland, CA). For each major biological question, a minimum of 3 was used per group, and at least 2 technical replicates (ie, 2 separate blots) were run in each case. Blots were stripped and reprobed with an antibody against total H3 to control for loading and nucleosome density. For quantification, band analysis was performed in ImageJ software (Open source package, Wayne Rasband, NIH). In the case of duplets (Fig. 5A), the top band was used for quantification. The resulting H3K9ac/H3 ratios for each blot were averaged across technical replicates and a *t* test was performed (2 tailed, nonparametric) on the values thus obtained for biological replicates.

### 2.8 RNA extraction

Frozen tissue was homogenized in 500 μL Trizol, separated into phases using chloroform and cleaned up with a Qiagen RNA microkit (74004; Qiagen, Manchester, UK) following manufacturer’s instructions. RNA quality was assessed on the Nanodrop (ThermoFisher Scientific, Loughborough, UK) for organic contamination and on the Agilent Bioanalyser (Agilent, Wokingham, UK) for degradation.

### 2.9 Chromatin immunoprecipitation

Frozen tissue was thawed and cross-linked in 1% formaldehyde for 10 minutes at room temperature. The reaction was quenched by the addition of 0.125 M glycine. The homogenates were lysed in 1% sodium dodecyl sulfate, 10 mM ethylenediaminetetraacetic acid, 50 mM Tris (pH 8.1), 1x Roche complete protease inhibitors and sonicated in a Diagenode bath sonicator (Bioruptor Diagenode, Liege, Belgium) to obtain an average chromatin shear size of 250 to 500 bp. Chromatin immunoprecipitation was performed as described before [57]. Briefly, the sheared chromatin was incubated overnight at 4 °C with protein G magnetic beads (Dynabeads; Invitrogen, Paisley, UK) and 1 μg of the appropriate primary antibody (anti-rabbit H3K9ac and anti-rabbit H3; Abcam). Rabbit immunoglobulin G was used as a negative control. Completed ChIP reactions were washed in standard wash buffers and Tris-EDTA (TE), before being de-crosslinked (0.2 M NaCl at 65 °C for 4 hours, followed by incubation with proteinase K for 1 hour). ChIP DNA was purified using Qiagen QIAquick PCR clean-up columns. One microliter of each ChIP sample was used for subsequent quantitative real-time polymerase chain reaction (qRT-PCR). Over the course of the entire protocol, care was taken to avoid bias or batch effects by processing treated and untreated samples in matched groups and always with their appropriate internal controls (H3, immunoglobulin G).

### 2.10 qRT-PCR

Ten microliters of standard qPCR reactions (Roche LightCycler480 mix, 0.5 μM of primers) were run on a Roche Lightcycler 480 system. All primers were validated with standard curves to test for appropriate efficiencies and melt curves, and their products were checked on a gel. For cDNA, the 2^ddCT method [48] was used with glyceraldehyde-3-phosphate dehydrogenase (Gapdh) as a control. For ChIP samples, standard curves were run on every plate to allow for absolute quantification.

The following primers were used (5′–3′):

Gene desert: *forward*_TGTAAGGGCCCTCCATGTAAA, *reverse*_ATACTGCATAGGCCACATCTTTC
Cacna2d1: *forward*_AAAAGCACCACAAACCCCT, *reverse*_TTTGCACAATCTGGCTGG
Hdac1: *forward*_TAGCCTTCCCTCCAGGAGTC, *reverse*_GGGTAGCCTGCGGTAATCTC
Hdac2: *forward*_ACACTTTCTTCTTGCCGCCT, *reverse*_CTCCCTCAGCCCTCTTGTCT
Hdac11: *forward*_TCCAACACAGTCCTCACAGC, *reverse*_TCCAGCCCTCTACACCCTAC
Rest: *forward*_TTAAAGCTCCACACGCACCT, *reverse*_GGGCCCAAGTTTGCAAAGAG
Mecp2: *forward*_TGCTCCCTCCTTCTCCTCTC, *reverse*_AGCTTCGAGTGCTGAGGAAC
Desmin: *forward*_CAAGTGGAGGTCCTCACCAAC, *reverse*_TTTCCTCCTGTAGCTTGGCCTT
Mt1a: *forward*_CCTCCTGCAAGAAGAGCTGC, *reverse*_AGCAGCACTGTTCGTCACTT
Mt2a: *forward*_TGCAGCGATCTCTCGTTGAT, *reverse*_AGCAGGATCCATCTGTGGCA
tRNA-cys: *forward*_GGGAGAAGCCTTAGTAGAGGAGA, *reverse*_GCCTTAAGGTGATTATCATGTCGAA
Gapdh: *forward*_ATGGGAAGCTGGTCATCAAC, *reverse*_CCACAGTCTTCTGAGTGGCA.

### 2.11 Microarray analysis

All tissue was taken from neuropathic animals, either drug or vehicle treated. RNA from 4 biological replicates per group was processed by UCL Genomics using an Ambion Whole transcript Expression Kit (Invitrogen) and hybridized to Affymetrix Rat Gene Arrays (Rat Gene 1.0ST) on a GeneChip Fluidics Station 450. Chips were scanned on an Affymetrix GeneChip Scanner. Quality control and subsequent analysis were carried out using the following bioconductor packages in R: *oligo* [10] for preprocessing, RMA normalization [34], and various quality controls (including MA plots, box plots, and principal component analysis), *limma* [55] for statistical analysis, and *biomaRt* [23] to access Ensembl v70 annotation. A recently characterized network reconstruction approach called causal reasoning was used to identify upstream regulators of any gene expression changes [14]. Causal reasoning uses directed molecular interactions (eg, protein X *increases expression of* transcript Y) to identify upstream regulators whose activity correctly explains a significant proportion of the observed gene expression changes. Importantly, causal reasoning takes into account the direction of transcriptional change (up or down) seen in the microarray data. Therefore, for a regulator to be significant, it must have both connections to differentially expressed genes and the expression data must also support the relationship (increases or decreases) between regulator and transcript. These criteria result in causal reasoning being robust to random noise [14] and can retrieve a signal where there is a consistent biological underpinning. The algorithm was applied by B.S. without previous knowledge of the specific HDACI compound used in this particular dataset (MS-275).

## 3 Results

### 3.1 HDACI reduced mechanical and thermal sensitivity after nerve injury

Vehicle or HDACI was delivered intrathecally to adult rats 5 days before and continuously throughout nerve injury–induced neuropathic pain. Two different class I HDACIs (MS-275 and MGCD0103) were used, and both were found to significantly attenuate mechanical hypersensitivity after injury by ∼40% to 50% (Fig. 1). This result was obtained across 4 independent experiments; in every one of them, the testing was carried out blind to experimental groups. Figure 1A shows a summary of all data, and Figures 1B and D show the effect of drug treatment on L5 spinal nerve transection and Figure 1C on partial sciatic nerve ligation. Group sizes ranged from 6 to 8 in individual studies, and results were statistically significant at a minimum of *P* < .05 (see Fig. 1 legends for more detail). Delivery of the HDACI before any insult had no effect on sensitivity thresholds, and the rats were ostensibly normal, with no obvious side effects.

In the L5 spinal nerve transection model, thermal sensitivity thresholds were also assessed (Fig. 3). The HDACI MS-275 significantly attenuated the injury-induced decrease in withdrawal thresholds in response to heat at both doses tested (*n* = 6, *P* < .05).

Pretreatment with HDACI before the insult was necessary for the observed analgesic effect to occur. When pumps were connected only after L5 spinal nerve transection, MS-275 could no longer affect mechanical thresholds (Fig. 4), suggesting that HDACIs, at least in this very specific paradigm, hold little promise as therapeutic targets.

### 3.2 HDACI reduced mechanical hypersensitivity in a more clinically relevant model of antiretroviral drug–induced neuropathy

Histone acetylation may also be involved in the generation of nontraumatic peripheral neuropathy. The antiretroviral drug d4T causes dieback of sensory axons from the periphery, reduces dorsal horn expression of calcitonin gene-related peptide and IB4 [32] and is often associated with severe and chronic neuropathic pain [9], [56], [62]. Some of the current authors (W.H. and A.R.) previously characterized d4T-induced neuropathy in the rat [32]. Using their model, the present study found that pretreatment and long-term administration of HDACI significantly increased hind limb withdrawal thresholds to punctate mechanical stimuli compared with vehicle controls (*n* = 7, *P* < .05) (Fig. 2). The magnitude of the effect was similar to that observed in nerve injury models, reducing the animals’ hypersensitivity to external stimulation by ∼40% to 50%.

### 3.3 HDACIs affected H3K9 acetylation in the spinal cord but not the dorsal root ganglia

**Fig. 6:**
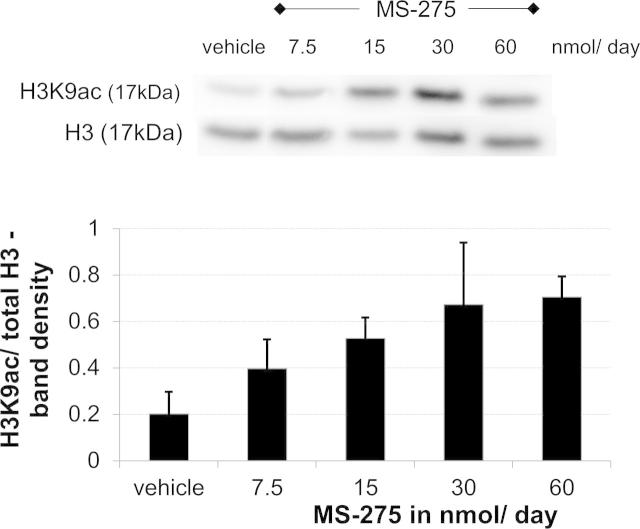
Intrathecal HDACi administration globally increased acetylation in rat spinal cord in a dose-dependent manner. MS-275 was administered to the spinal cord at increasing doses (7.5–60 nmol/d), the protein extracted from dorsal lumbar segments and probed with an antibody against the acetylated lysine residue 9 of histone 3 (H3K9ac). Shown here is a representative Western blot with its corresponding loading control against histone H3. Quantification of 2 separate blots with several biological replicates (*n* = 2–3) was performed using band-density analysis in ImageJ software. Data are expressed relative to H3.

As expected, intrathecal treatment with MS-275 and MGCD0103 increased spinal acetylation of histones due to the HDACs not being able to exert their effect. Using Western blot, global acetylation was measured at lysine residue 9 and found to be significantly increased in injured dorsal lumbar cord after drug administration. The effect was dose dependent (Fig. 6), reaching a ceiling at 30 nmol/d and could be observed in both traumatic and drug-induced models (Fig. 5).

**Fig. 7:**
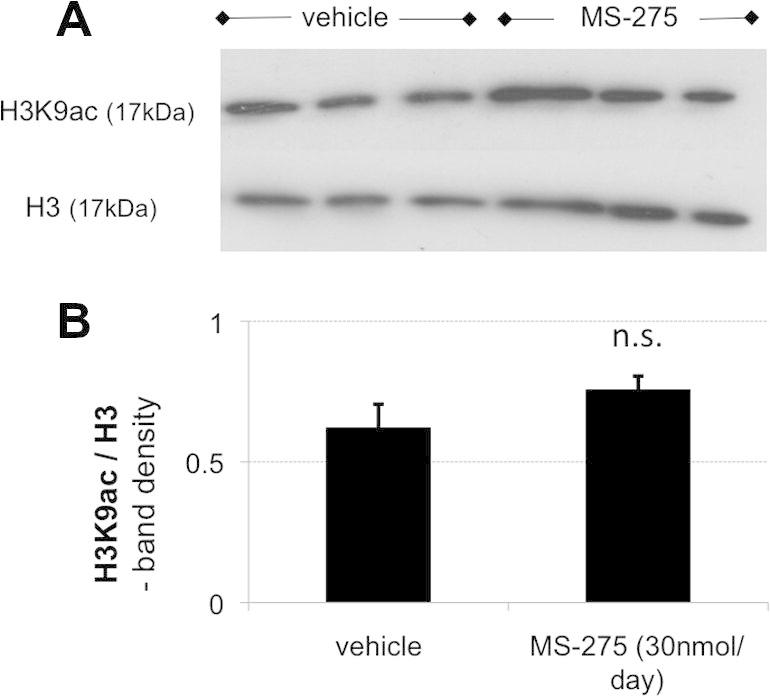
With the intrathecal delivery method used, HDACI did not appear to have a measurable effect on dorsal root ganglion (DRG) acetylation, suggesting that the mechanism of action was mostly central. (A) Representative Western blot of single rat L5 DRG after nerve transection and intrathecal vehicle or HDACi treatment. Total H3 was used as a loading control for acetylated H3K9. (B) Quantification of global H3K9ac revealed no difference between vehicle and HDACI treatment groups (*n* = 4, *P* = not significant [n.s.]).

**Fig. 8:**
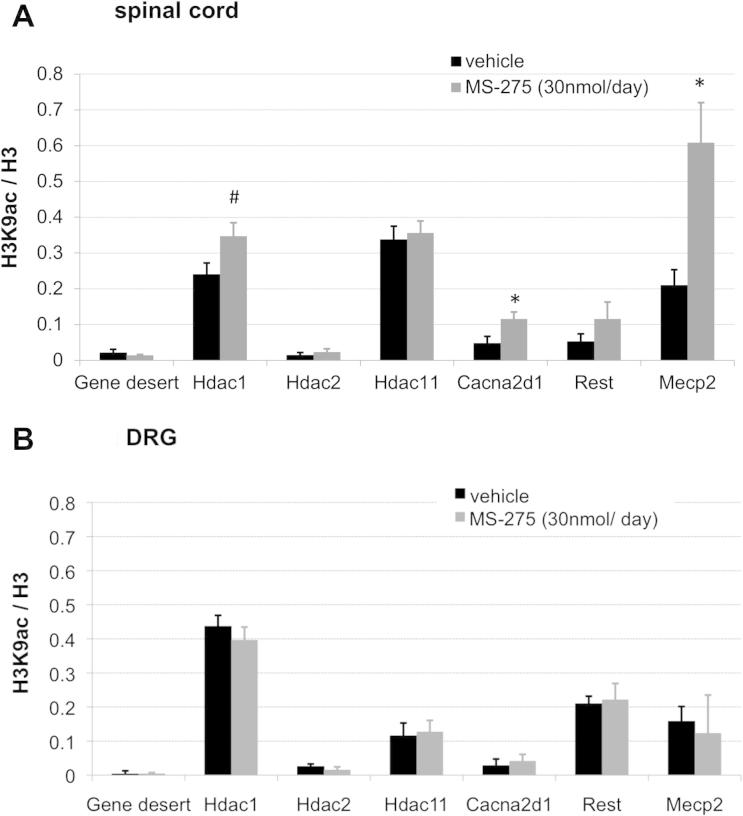
ChIP–quantitative polymerase chain reaction (qPCR) revealed changes in H3K9ac with drug treatment at several promoters in the spinal cord, but not the dorsal root ganglia (DRG). (A) ChIP-qPCR of dorsal ipsilateral spinal cord tissue examining H3K9ac at relevant promoters. Shown here is the ratio of enrichment of H3K9ac over H3 for a gene desert control region and the transcriptional start sites of 6 genes: Hdacs1, 2, and 11; the calcium channel subunit α2δ1 (Cacna2d1), and the transcription factors Rest and Mecp2. Histone deacetylase inhibitor resulted in increased enrichment at 4 of 6 of the genes tested, statistically significant in the case of Cacna2d1 and Mecp2 (*n* = 4, independent-sample *t* tests, uncorrected *P* = .016, *P* = .047, respectively). (B) In the DRG, MS-275- and vehicle-treated ipsilateral DRG revealed no significant differences between treatment groups at any of the genes tested. ^∗^*P* < 0.05, ^#^*P* < 0.1.

Interestingly, the drugs did not appear to reach the dorsal root ganglia (DRG) relevant for hind limb sensitivity (L4–6) at detectable concentrations, possibly due to high catheter placement. Thus, Western blots of L5 DRG showed no clear difference in acetylation between drug-treated and nondrug-treated tissue (Fig. 7). Equally, examining H3K9 acetylation at individual promoters revealed increases only in spinal cord, but not L5 DRG tissue (Fig. 8).

Six genes were chosen for testing with ChIP-qPCR: the 2 most prominent class I HDACs (Hdac1 and Hdac2); Hdac11, which should not be affected by MS-275 [7]; the calcium channel subunit α2δ1 (Cacna2d1), as one of the genes known to be greatly transcriptionally dysregulated after neuropathic injury [21]; and 2 transcription factors that have been implicated in the epigenetic regulation of pain conditions (Rest and Mecp2 [27], [59]). None of them displayed significant acetylation changes at lysine residue 9 in the DRG (Fig. 8B), although clear changes could be observed in the spinal cord (Fig. 8A). Independent *t* tests were statistically significant for Cacna2d1 (*P* = .047) and Mecp2 (*P* = .016) and nearly significant for Hdac1 (*P* = .074). Considering the high variability associated with the ChIP-qPCR technique and the very heterogeneous cell populations of the spinal cord, differences of this magnitude are unlikely to be due to type I errors, even though they did not survive stringent Bonferroni correction.

Finally, microarray analysis of RNA extracted from HDACI or vehicle treated, injured L5 DRG was undertaken. Ipsilateral injured DRG were analyzed in 4 biological replicates per group. All RNA samples and Affymetrix arrays passed quality control. However, the microarrays revealed no significant changes in expression after multiple comparison corrections. Even at a very permissive threshold, of unadjusted *P* < .01, only 8 genes showed any change in expression above 1.5-fold (Table 1 and Supplementary Table for full list of genes). Pathway analysis was conducted using a recently characterized network approach called causal reasoning (see Section 2 and Ref. [14]). The algorithm is more robust to random noise because it identifies upstream regulators of differentially expressed genes and takes into account the direction of molecular interactions. Despite this, no regulators or networks emerged as significant in the DRG that could conceivably be related to abnormal HDAC activity (data not shown).

**Table 1:** Summary counts of differentially expressed genes in ipsilateral dorsal cord and ipsilateral DRG at 2 levels of stringency: an FDR-corrected *P* value < .05 and an uncorrected *P* value < .01.

Stringency		Down	No change	Up
FDR correction, *P* < .05	Spinal cord	0	29,213	Mitochondrial transfer RNA ENSRNOG00000033932 4.55x upregulated
	DRG	0	29,214	0

No FDR correction, *P* < .01				
At any fold change	Spinal cord	180	28,835	199
	DRG	103	29,051	60
At fold change ⩾ 1.5	Spinal cord	12	29,184	18
	DRG	4	29,206	4

DRG, dorsal root ganglia; FDR, false discovery rate.

*Note:* Microarray analysis of RNA extracted from histone deacetylase inhibitor– or vehicle-treated injured tissue (*n* = 4) revealed only a single gene that survived multiple testing corrections: a mitochondrial transfer RNA in the spinal cord with EnsemblID ENSRNOG00000033932. At a very permissive threshold of uncorrected *P* < .01, a small number of genes showed significant changes: 379 in the spinal cord and 163 in DRG. Due to the potential for false positives in this list, these genes were only used for causal reasoning analysis, which is robust to random noise.

Taken together these results suggest that, with the specific intrathecal delivery protocol described, HDACIs most likely exerted the majority of their effects centrally at the level of the spinal cord.

### 3.4 Microarray analysis of spinal cord mRNA exposed the specific signature of MS-275 and suggested that the drug’s transcriptional effect was mediated through HDAC1

In contrast to the DRG, microarray analysis of the spinal cord after nerve injury offered a more interesting picture. RNA from dorsal ipsilateral lumbar segments, treated with vehicle or HDACI, was hybridized to Affymetrix microarrays (*n* = 4). Again, all chips passed standard quality controls, but principal component analysis showed little separation between groups. Unlike in the DRG, this was a less surprising outcome. Even with drastic interventions such as nerve injury or complete Freund’s adjuvant, research commonly only reports small fold changes (<2) in the spinal cord, most likely due to high tissue heterogeneity [28], [39], [53].

Only a single gene passed standard false discovery rate correction (FDR) at *P* < .05: ENSRNOG00000033932, a mitochondrial cysteine transfer RNA (tRNA-cys), significantly upregulated after HDACI treatment. A larger, but still modest list of nominally significant genes was identified at uncorrected *P* < .01 (Table 1 and Supplementary Table), a threshold at which false positives can undoubtedly pass. Again, therefore, genes were not examined individually, but analyzed further using causal reasoning to retrieve signals with consistent biological underpinning. Of the nominally significant probes, 177 were annotated with an Ensembl ID and had an unequivocal ortholog in humans. Of those, 83% were present in the knowledge base of causal interactions that forms the basis for the causal reasoning algorithm.

**Fig. 9:**
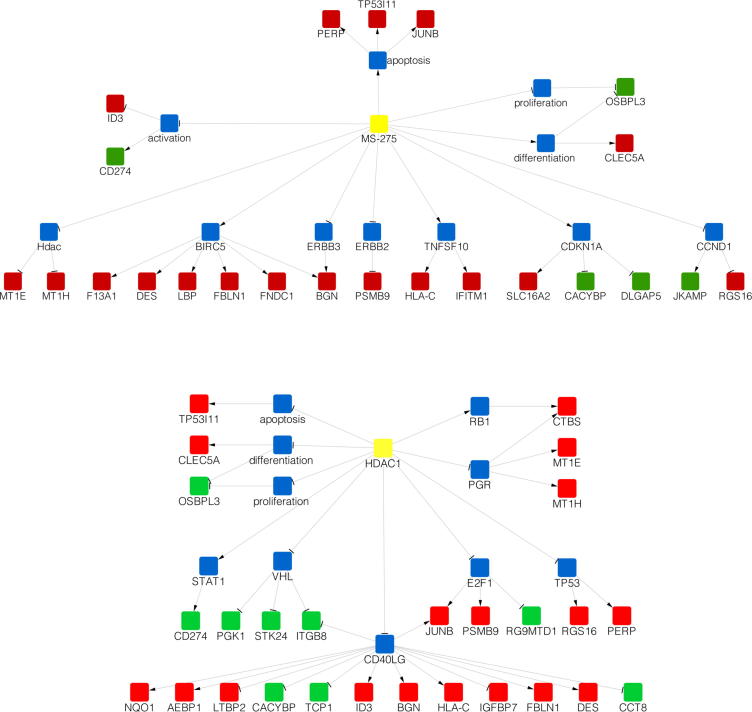
Causal reasoning applied to spinal cord microarray data revealed an MS-275 signature and suggested that the effect of this particular HDACI was mediated through HDAC1. Shown here are the causal networks for MS-275 (A) and HDAC1 (B). Yellow nodes represent the upstream regulators. Red and green nodes are genes in the nominally significant list of transcripts that were observed to increase or decrease in expression, respectively. Blue nodes are intermediaries introduced by the algorithm and can be either proteins or biological processes. The arrow and bar edge endings indicate an activating or repressive relationship, respectively. Genes chosen for further validation were MT1E (rat ortholog Mt1a), MT1H (rat ortholog Mt2a), and desmin.

The top causal hypothesis that emerged from the analysis was for an increase in the compound MS-275 (Bonferroni corrected *P* = .0026), which could be connected to 28 of the 177 genes (Fig. 9 and Table 2). This means that the changes observed in the microarray analysis matched previously reported changes induced by MS-275 and can be said to carry the “signature” of this particular HDACI. A reassuring result-demonstrating that causal reasoning could successfully discriminate relevant pathways from any noise. Of further interest in the top 10 causal regulators was the hypothesized decrease in HDAC1, which could be connected to 32 genes, 27 of them with correct directionality. HDAC1 is one of the primary targets of MS-275, and these results suggest that a significant proportion of the expression changes observed were due to its impaired function after drug administration.

**Table 2:** MS-275 and HDAC1 emerged as mediators of gene expression changes using causal reasoning on 177 nominally significant genes.

Hypothesis	*P* (Bonferroni)	Enrichment	C	I	A	Type
MS-275+	2.63E−06 (0.029)	1.81E−05	21	5	2	Chemical
Roscovitine−	1.27E−05 (0.141)	3.04E−05	19	5	5	Chemical
HDAC1−	1.42E−04 (1.569)	8.81E−03	19	5	8	Protein
DHEA sulfate−	1.34E−03 (14.848)	8.77E−05	26	12	7	Chemical
SMAD3+	2.06E−03 (22.797)	1.81E−04	26	12	10	Protein
TLR2+	3.44E−03 (38.013)	4.86E−03	28	14	12	Protein
LY294002−	4.22E−03 (46.638)	3.60E−03	31	16	23	Chemical
ADRB2+	4.34E−03 (47.967)	9.56E−04	29	15	12	Protein
SNCA+	8.07E−03 (89.172)	1.62E−03	28	15	12	Protein

A, number of ambiguous connections; C, number of causally correct paths from the hypothesis to the differentially expressed genes; DHEA, dehydroepiandrosterone; HDAC, histone deacetylase inhibitor; I, the number of incorrect paths.

*Note:* The sign in the hypothesis column indicates whether an increase (+) or decrease (−) in the activity of the hypothesis is predicted to cause the observed expression changes. The *P* column contains the unadjusted *P* values, with Bonferroni-adjusted *P* values in parentheses. This test takes into account the directionality of the fold changes. In contrast, the Enrichment column *P* values reflect the proportion of downstream regulators present in the gene expression dataset.

**Fig. 10:**
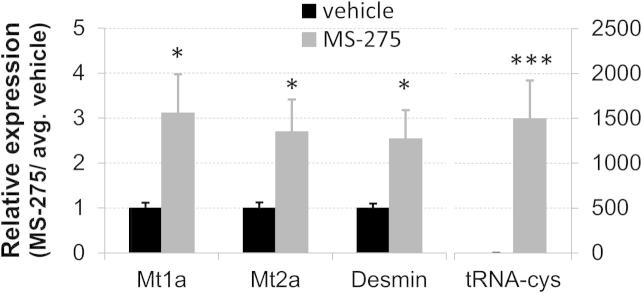
Quantitative real-time polymerase chain reaction (qRT-PCR) validation of spinal cord microarray data confirmed increased expression of (HDAC)1 targets and of a mitochondrial transfer RNA. Primers were designed against HDAC1 targets (2 metallothioneins [Mt1a, Mt2a, human orthologs: MT1E, MT1H] and desmin) as well as the cysteine transfer RNA (tRNA-cys) identified via microarray. All values were normalized to Gapdh (2^-ddCT), and relative expression values were calculated in relation to the vehicle average. Increased transcription was observed in drug-treated tissue, highly significant in the case of tRNA-cys: uncorrected *P* values of .027 (Mt1a), .026 (Mt2a), .047 (desmin), and .00013 (tRNA-cys); independent-sample *t* tests, *n* = 4.

To validate some of these microarray results, qRT-PCR was carried out on an independent cDNA batch to test expression of 3 downstream targets of HDAC1 action, as well as the significantly dysregulated tRNA-cys (Fig. 10). Two metallothioneins, Mt1a and Mt2a, and the intermediate filament desmin showed a 2.5- to 3-fold increase in expression, further supporting a small, but robust effect on HDAC1 target genes. Equally consistent with the array data, tRNA-cys was increased 1500-fold in HDACI-treated tissue, representing a highly significant increase at *P* = .00013.

## 4 Discussion

The data presented provide the first direct evidence of a role for histone deacetylases in the development of neuropathic pain conditions. Delivery of HDACI into rat spinal cord was found to reduce hind paw hypersensitivity by ∼40% in several different models of neuropathic pain: partial sciatic nerve ligation, L5 spinal nerve transection, and a systemic model of antiretroviral drug–induced neuropathy with quite different progression and neuropathology [32]. These results are in line with recent work reporting analgesic effects of HDACI in acute inflammatory models [2], [13], [65].

In contrast, drug treatment did not affect already established neuropathic pain after spinal nerve transection, suggesting that histone acetylation might be specifically involved in the emergence of hypersensitivity. To dissociate this further, future experiments could be conducted in which drug pretreatment is stopped at the time of injury. It also cannot be completely ruled out that an effect might appear at a higher dose, even though the threshold for full global hyperacetylation appeared to have been reached at the 30-nmol/d dose used in this study.

On a mechanistic level, it seems likely that HDACIs exerted their effect centrally within the spinal cord. No evidence could be found for involvement of the DRG, with the compounds having no clear effects on injured tissue in terms of global histone acetylation, local promoter acetylation, or RNA expression levels. This does not necessarily mean that neuropathic pain processes in the DRG cannot be modulated by epigenetic mechanisms, but only that they were not affected in this particular case, most likely because catheters were placed optimally to treat the lumbar spinal cord and therefore were relatively far from L5 ganglia.

In contrast, at the level of the spinal cord, HDACI significantly increased H3K9 acetylation and altered mRNA expression. H3K9ac levels were affected both globally (as measured by Western blot) and locally at various relevant promoters (as measured by ChIP). Microarray analysis revealed a mitochondrial transfer RNA as significantly upregulated in drug-treated tissue. Moreover, the expression pattern of a nominally significant list of genes was consistent with an MS-275 signature as well as a decrease in HDAC1 function.

Of note, MS-275 did not directly affect acetylation at all promoters tested, despite its selectivity for the ubiquitous class I HDACs [7]. Of 6 genes tested, changes were observed in 4 (Hdac1, Cacna2d1, Rest, Mecp2), 2 of which were statistically significant at *P* < .05: Mecp2 and Cacna2d1. Mecp2 (methyl-CpG-binding protein) methylates DNA and can thus lead to transcriptional silencing. There is evidence to suggest that MeCP2 might act as a global regulator of chromatin structure, its joint presence with HDACs in repressor complexes enabling it to influence acetylation and allowing a link between acetylation and DNA methylation patterns [54]. The increased acetylation at MeCP2 could therefore be the system’s attempt to counterbalance diminished HDAC action after drug administration.

The functional effect of increased H3K9ac at the calcium channel subunit Cacna2d1 is less clear. The gene is known for abnormal increases in transcription after neuropathic pain [21], and the microarray data presented here suggest that these were unchanged with HDACI administration. Hence, in this case, local H3K9 acetylation clearly failed to correlate with transcription. It could be that at the Cacna2d1 promoter, H3K9ac does not have any real biological significance: the overall enrichment was very small, barely exceeding the negative control levels of a gene desert region. The study of other marks posited to correlate with transcription, such as H3K27ac and H3K4me3 [22], might be more revealing. Either way, HDACIs are unlikely to exert their effect by reversing the abnormal transcriptional regulation of Cacna2d1.

Generally, the measurable effect of HDACI treatment on transcription was modest, although the emergent MS-275 signature suggested that expected changes did occur, and causal reasoning further suggested that they were mediated through decreased HDAC1 function. This was also confirmed by qRT-PCR, which showed increased expression of several HDAC1 targets.

MS-275 administration did not affect HDAC1 itself at day 14 (fold “change” of 1.06). It could be that an earlier effect on transcription was missed, although a different explanation seems more likely given what is known about HDACI function. These compounds block access of histone lysine residues to the active site of HDACs [25], and their effects may differ depending on which particular HDACI protein complex they are incorporated into [3]. In this case then, MS-275 may have blocked HDAC1 from accessing and binding to its usual targets, affecting expression of a variety of genes downstream. Perhaps the hint of increased H3K9 acetylation observed at the HDAC1 promoter itself could be interpreted as the system’s attempt to counteract the drug-induced blockade of HDAC1 function.

Only a single gene survived multiple comparison corrections and was significantly changed in MS-275 treated tissue: a mitochondrial cysteine transfer RNA, the relative expression of which was increased by nearly 5-fold in the microarray and 1500 times in subsequent qRT-PCR. Transfer RNAs are essential for the translation of specific amino acids, in this case of cysteine in the mitochondrial genome [52], [64]. There appears to be no literature linking these RNAs to HDACIs, histone acetylation, or chronic pain. There is a putative link between mitochondrial stress and the development of chronic pain conditions, such as chemotherapy-induced neuropathy [26], [63]. Moreover, in the d4T model specifically, it has been shown that abnormal regulation of mitochondrial proteins might contribute to the degeneration of distal axons [32]. Yet, it still remains to be seen whether any of this relates specifically to transfer RNAs and hence whether there could be a real biological connection between this specific cysteine-tRNA and neuropathic pain.

In summary, only limited effects of HDACI on transcription were observed in this study, which might be due to the mix of cell types and the complexity of the systems involved. HDAC inhibition is often simplistically equated with hyperacetylation and hence increased transcription. Yet, the regulation of histone acetylation is a very intricate, highly cell type–specific process involving not only HATs and HDACs, but also multiple other cellular proteins and possibly even microRNAs [33], [50].

HDACs themselves are a large family of 4 classes: zinc-dependent classes I, II, and IV and the biochemically quite distinct class III or sirtuins. Although class I HDACs (HDAC1–3) are ubiquitously expressed, they are currently the only zinc-dependent HDACs that inhibitors can convincingly target in a select fashion [7] and were therefore chosen for this study. HDAC4, HDAC5, and HDAC11 also show high expression levels in the central nervous system [8], but their function would have to be elucidated with genetic or transgenic approaches.

There is convincing evidence from work on yeast and T cells that HDACs can be associated with gene activation as well as repression [37], [60], and HDAC function is thought to be very dynamic and context dependent [4], [17]. Deacetylase enzymes, in particular classes II and III, also act on cytoplasmic proteins and are thus able to influence a wide range of processes including translation, microtubular transport, and membrane regulation [45].

Perhaps as a consequence of this, HDACIs are reported to have differential impact on H3K9 acetylation depending on promoter status (active, poised, or silent) [60] and are known to have relatively modest effects on transcription [30], [44], possibly due to a limited supply of transcriptional machinery [54]. These findings, combined with the generally small fold changes observed in highly heterogeneous spinal cord tissue [28], [39], lead one to expect difficulties in teasing out biologically relevant expression changes after HDACI treatment. Technical advances, such as cell sorting techniques like flow cytometry, could help resolve some of these issues in the future.

It remains to be seen whether nerve injury itself changes histone acetylation or indeed other modification marks in the spinal cord. Given that HDACI can positively affect hypersensitivity in the models described here, this possibility certainly presents itself as a major hypothesis, albeit a challenging one to explore. Addressing it directly will most likely require genomewide data from ChIP-seq and, even more so than in the case of transcription, thorough cell type–specific analysis.

### 4.1 Conclusions

This study has explored the potential involvement of an epigenetic process in chronic neuropathic pain. Although the precise mechanisms still remain to be determined, the resulting data strongly suggest that chemical interference with histone acetylation at the level of the spinal cord can ameliorate the sensory abnormalities associated with both traumatic nerve injury and drug-induced neuropathy.

## Conflict of interest statement

B.S. and D.Z. are full-time employees of Pfizer Ltd. None of the other authors have any conflicts of interest to declare.
